# Immunotherapy in mucosal melanoma: a case report and review of the literature

**DOI:** 10.18632/oncotarget.24727

**Published:** 2018-04-03

**Authors:** Hana Studentova, Hana Kalabova, Pavel Koranda, Karin Chytilova, Ladislava Kucerova, Bohuslav Melichar, David Vrana

**Affiliations:** ^1^ Department of Oncology, Palacký University, Faculty of Medicine and Dentistry and University Hospital, Olomouc, Czech Republic; ^2^ Department of Nuclear Medicine, Palacký University, Faculty of Medicine and Dentistry and University Hospital, Olomouc, Czech Republic; ^3^ Department of Maxillofacial Surgery, Palacký University, Faculty of Medicine and Dentistry and University Hospital, Olomouc, Czech Republic; ^4^ Department of Pathology, Palacký University, Faculty of Medicine and Dentistry and University Hospital, Olomouc, Czech Republic; ^5^ Institute of Molecular and Translational Medicine, Palacký University, Faculty of Medicine and Dentistry and University Hospital, Olomouc, Czech Republic

**Keywords:** mucosal melanoma, head and neck, immunotherapy, ipilimumab, late response

## Abstract

**Background:**

Mucosal melanoma is a rare form of melanoma presenting variably as sores or unexplained bleeding located mainly in the head and neck region, anorectal region or female genital tract. Mucosal melanoma is usually diagnosed at an advanced stage and is characterized by an aggressive behavior. Surgery represents the mainstay of treatment for early stage melanomas, but for advanced disease there have been until recently very limited treatment options. Ipilimumab, a human monoclonal antibody directed against the cytotoxic T lymphocyte antigen 4, was the first treatment modality to demonstrate survival benefit in advanced malignant melanoma.

**Method:**

Description of a new case and review of the literature.

**Results:**

We present here a patient with mucosal melanoma with aggressive biological behavior and documented late response to ipilimumab.

**Conclusions:**

Ipilimumab represents an effective treatment option in selected patients with mucosal melanoma.

## INTRODUCTION

Mucosal melanoma is a rare form of malignant melanoma, comprising only about 1% of all melanoma cases. Compared to the cutaneous primary mucosal melanomas have clinically and biologically distinct pattern of behavior characterized by aggressive growth with high rate of locoregional recurrence and distant metastasis as well as 5-year disease-free survival rates ranging only between 0 and 20% [1–3]. Approximately 50% of mucosal melanomas arise from the mucosa in the head and neck region, with most of the other cases involving anorectal and vulval or vaginal mucosa. Unlike cutaneous melanomas, mucosal melanomas are more prevalent in older women and more equally geographically distributed [2]. Surgical resection with adequate margins is the optimal treatment strategy in localized mucosal melanoma, but this strategy is often not possible, and relapse rates after surgery are high [3]. Regarding genetic profile, BRAF mutations have been detected only in about 10% [4] compared to 50% in cutaneous melanoma. On the other hand, c-kit gene aberrations have been found more frequently, in about 16–25% of mucosal melanomas compared to only 5–10% in cutaneous melanomas [4–6]. NRAS mutations were detected in 25% mucosal melanoma cases [5].

Since the occurrence of mucosal melanoma is rare, it is difficult to conduct prospective randomized trials to evaluate potential treatment modalities in patients with malignant melanoma of this primary site. Thus, only limited published data on the efficacy of therapeutic options in the treatment of mucosal melanoma are available. Currently, immunotherapy represents the mainstay of therapy in patients with BRAF wild-type metastatic melanoma.

Ipilimumab (Yervoy^®^, Bristol-Myers Squibb, New York, NY, USA) is a human monoclonal antibody targeting cytotoxic T-lymphocyte antigen 4 (CTLA-4). After confirmation of survival benefit in two phase III trials in patients with cutaneous melanoma [7, 8], ipilimumab was approved by U.S. Food and Drug Association in March 2011. Systemic therapy for melanoma of other primary locations, including mucosal melanoma remains undefined since no prospective clinical trial has been published so far, and only limited retrospective data exist [9]. Thus, it remains unclear whether immunotherapy has the same efficacy as in patients with cutaneous primary, although in the clinical practice the treatment is selected based on data obtained in patients with malignant melanoma.

We present here an exceptional case of a patient with mucosal melanoma characterized by highly aggressive behavior with delayed partial response to ipilimumab.

## CASE REPORT

**Figure 1:**
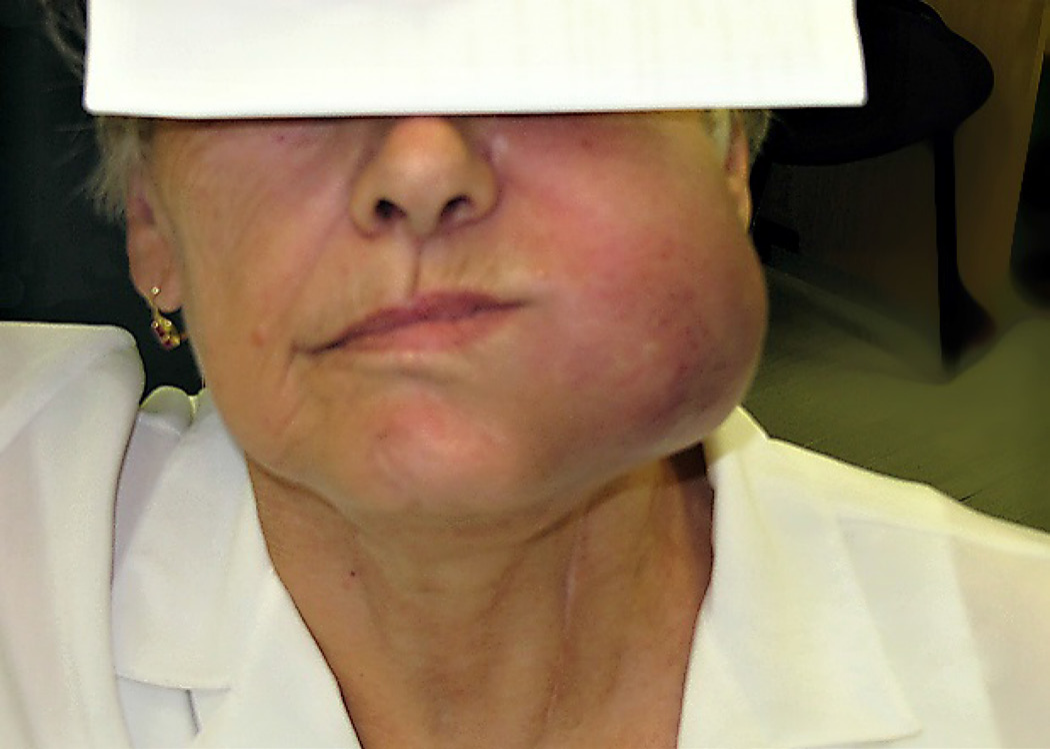
Primary mucosal melanoma of the left upper alveolus before ipilimumab therapy initiation

**Figure 2:**
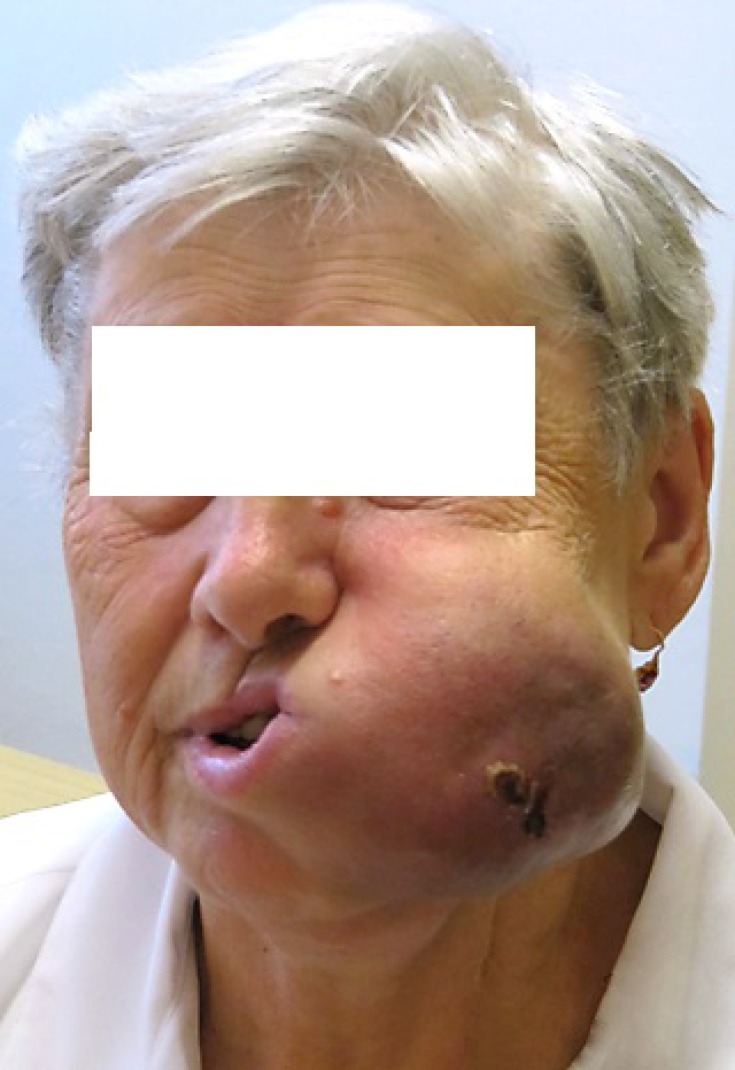
Rapid tumor progression during ipilimumab therapy

**Figure 3:**
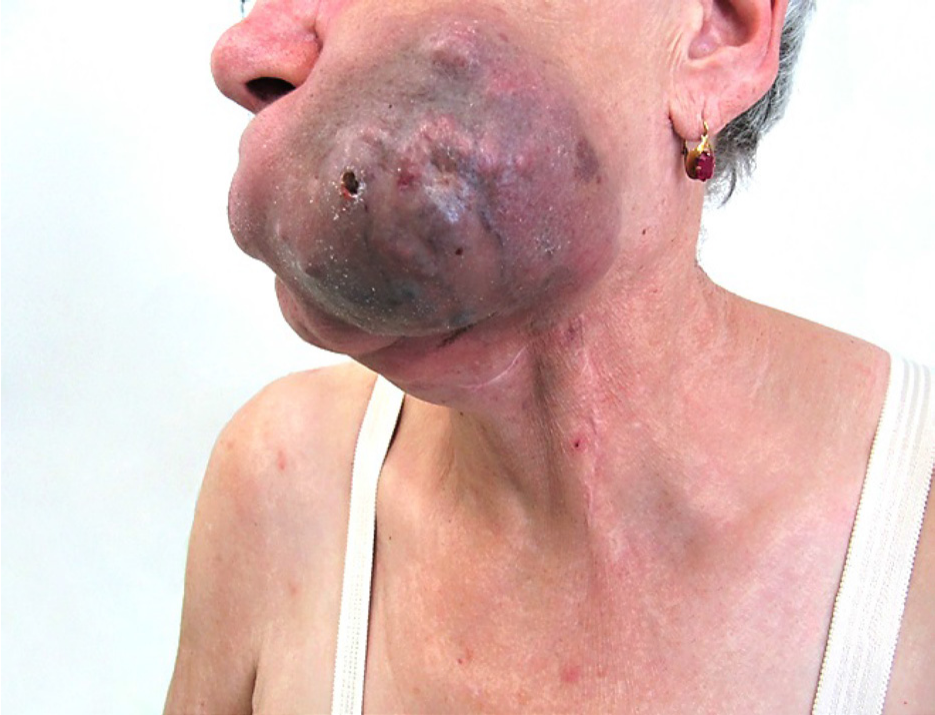

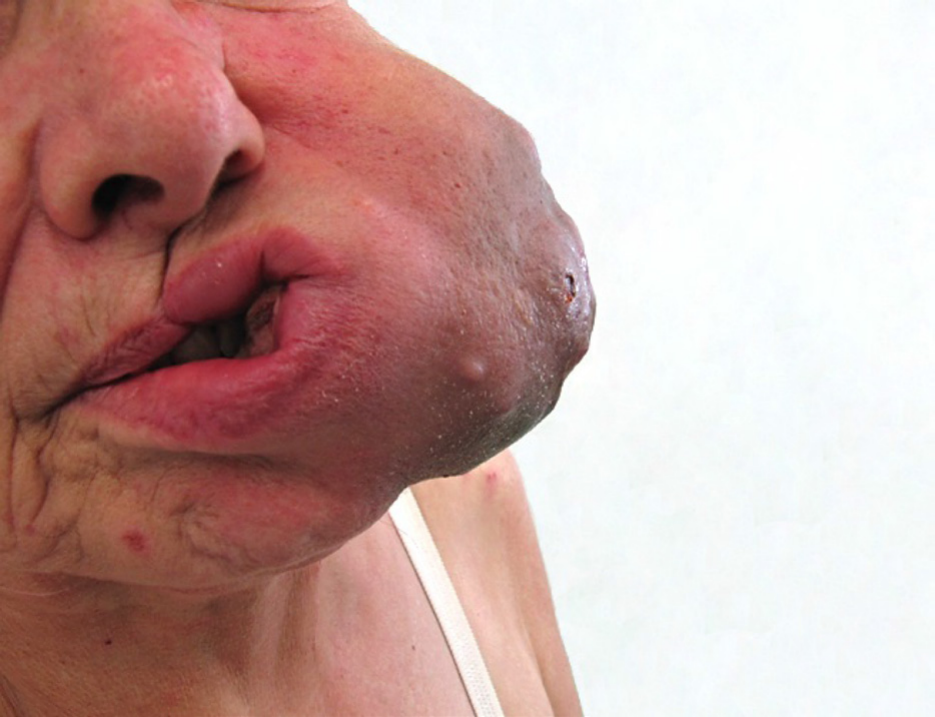
Continuing tumor progression (**A**) lateral view, (**B**) front view.

**Figure 4:**
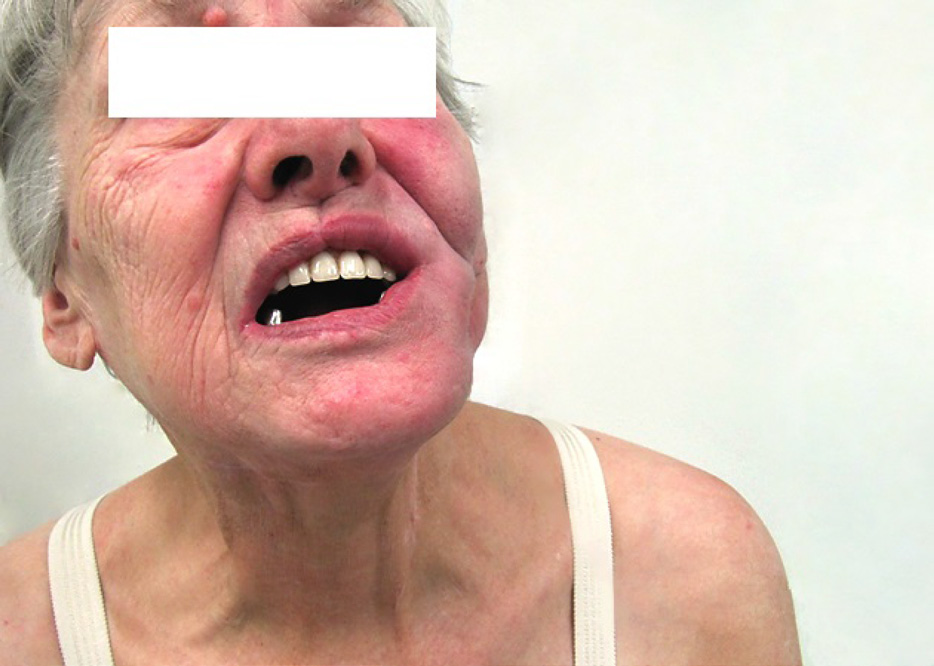
Complete regression of the primary tumor (38 weeks from ipilimumab initiation)

A 69-year-old woman with no significant comorbidity presented in April 2011 with a recurrent sore on her left upper alveolus. Based on histological examination of an excision biopsy, diagnosis of mucosal malignant melanoma was confirmed. Whole body [^18^F] fluorodeoxyglucose (FDG) positron-emission tomography/computed tomography (PET/CT) did not detect metastatic spread. In May 2011, the patient was referred for radical surgical procedure with wide surgical resection of the palate and submandibular salivary gland resection and bilateral cervical lymphadenectomy. Clear pathological margins were obtained, with the lesion 15 mm in length with 5 mm invasion, and lymph nodes unaffected. After healing of surgical wounds, the patient underwent adjuvant radiotherapy consisting of 30 Gy delivered to the tumor bed and left submandibular lymph nodes in 6 Gy fractions. In February 2012, a local recurrence of the tumor had to be removed again, but within a month, a new tumor recurrence manifested in the same location. Having realized no further sense of surgical intervention, the patient was offered participation in a clinical trial with ipilimumab. On initial CT scan, apart from rapidly growing local tumor recurrence (Figure 1), multiple pulmonary metastases were identified. The tumor was BRAF wild type. At the initiation of ipilimumab treatment in June 2012, serum level of lactate dehydrogenase (LDH), absolute lymphocyte count (ALC) and C-reactive protein (CRP) were 2,03 μkat/L (normal range 2, 25–3, 55 μkat/L), 2900/μL (normal range 800–4000/μL) and 56, 3 mg/L (upper limit of normal is 5 mg/L), respectively. The patient received ipilimumab at the dose of 10 mg/kg every 3 weeks for four doses as per protocol of the clinical trial. The patient had progressive disease (PD) as assessed by immune-related Response Evaluation Criteria In Solid Tumors (irRC) at the time of the first radiographic assessment (after two doses of ipilimumab) (Figure 2), but the treatment was continued as per protocol and in August 2012, all four cycles of the treatment were completed. Following continuing tumor progression (Figure 3) with significant disability of food intake, we faced therapeutic despair and decided to treat the patient with chemotherapy. The patient received two cycles of systemic dacarbazine at the dose of 1000 mg/m^2^ in a 3 week schedule, but the treatment was terminated because of further progression. The patient was discharged to home care with referral to hospice. Surprisingly, the patient came to the clinic in February 2013 and the tumor in the head region disappeared (Figure 4). The CT scans demonstrated significant tumor regression showing complete response of the mucosal melanoma in the mouth and only residual lung metastases were evident. Ipilimumab therapy was not associated with any serious adverse reaction, only vitiligo appeared several months after the therapy. After 8 months of the disease being stabilized, progression in the lungs was observed followed shortly by local recurrence. Discussing further patient treatment, we attempted to obtain ipilimumab for reinduction, but ipilimumab was not reimbursed in the reinduction setting in the Czech Republic. Moreover, the study protocol did not allow to retreat the patient with ipilimumab unless there was partial response as the overall response assessed during the clinical trial, but the patient was discontinued from the clinical trial because of disease progression. We had the tumor sample re-examined again and it was found to be c-kit negative, but positive for PDGFRA mutation in exon 18.

Because of poor performance status the patient was subsequently treated only symptomatically and died on January 26, 2014.

### Discussion and review of the literature

We report here an exceptional case of mucosal melanoma patient with rapid tumor progression of the primary tumor. Following continuous disease progression after surgical resections, the patient was treated with ipilimumab monotherapy that was initially followed by disease progression, but subsequently by disappearance of the primary tumor and overall partial response of the disease elsewhere 8 months later. However, the effect lasted only for another 8 months and disease progression occurred followed by death 3 months later.

Treatment options for patients with mucosal melanomas are limited. Although the systemic therapy is suggested to be less effective than in cutaneous melanoma, because of the lack of specific data patients with mucosal melanomas are usually treated with the same regimens. Given the rarity of the disease, results of registration trials in most frequent patient subgroup of cutaneous melanoma may not address questions of daily routine practice such as efficacy of the treatment in patients with mucosal primary. In these patients retrospective series, usually limited to few patients, or even case reports like the present case are the only source of information on patient management.

Due to the low incidence of mucosal melanoma only limited data have been published regarding the efficacy of therapeutic options. Yi *et al.* reported that among patients with non-cutaneous melanoma mucosal primary is a poor prognostic factor for the outcome of treatment with dacarbazine-based chemotherapy regimens [10]. In a single-institution series on biochemotherapy in mucosal melanoma using the combination of cisplatin, vinblastin, dacarbazine, interleukin-2 or interferon-alpha response rate between 36 and 47% was observed, but these studies were small with number of patients ranging from 11 to 18 [11–13]. Since immunotherapy currently represents the mainstay of treatment for the majority of patients with metastatic melanoma, there are also some retrospective data on the outcome of ipilimumab treatment in patients with mucosal melanoma. Due to the limited patient numbers, it may be difficult to interpret the data. Most information on this topic comes from patients with mucosal melanoma treated in several expanded access programs (EAPs) [14–17]. According to some reports, response rates are similar to previously reported data in ipilimumab treated patients with cutaneous melanoma [7, 18, 19]. Postow *et al.* in a multi-institutional retrospective analysis reported an immune-related best overall response rate (irBORR) of 6.7% with an immune-related disease control rate (irDRC) of 23.3%. Although the response rate was relatively low, ipilimumab induced some durable antitumor effects in individual patients [20]. Similar results were reported from an Italian EAP by delVecchio in the largest study evaluating 71 patients with mucosal melanoma [17]. However, the median overall survival of 10.1 and 11.2 months observed in ipilimumab registration trials seems to be longer compared to median overall survival reported in smaller trials in mucosal melanomas of 6.4 , 6.7 and 5.8 months, respectively [14, 17, 20]. DelVecchio *et al.* reported irDRC of 36% and irBORR of 12% which is higher than in the US series [17, 20], nevertheless irSD was far more common than reduction in tumor burden which is a typical pattern of response to ipilimumab and is associated with prolonged survival [19, 21]. Zimmer *et al.* reported an open-label, multicenter, single-arm phase II DeCOG-study of ipilimumab in pretreated patients with different subtypes of metastatic melanoma [22]. In this trial, 7 patients with mucosal melanoma were treated, out of whom only 4 completed the induction phase with 4 cycles of ipilimumab. For mucosal melanoma, the 1-year OS rate in this study was 14% (95% CI 1–47) and all patients died within 24 months after the first ipilimumab dose. Out of 6 evaluable patients, one achieved partial response (PR) and two had disease stabilization (SD).

Similarly to the present case, there have been cases of late responses to immunotherapy described in the literature [23, 24] followed by durable antitumor effect, in some cases resulting in complete responses [24] , with the tumor considered to be potentially cured. It is well known that building an immune response against any solid tumor can take time with initial irSD resulting in irPR or even irCR after some time from immunotherapy initiation [24]. To the best of our knowledge, similar pattern of response to ipilimumab has been described in patients with rapidly progressing mucosal melanoma only in another single case report [25].

Monoclonal antibodies targeting PD-1/PD-L1 interaction have been shown to be more effective compared to ipilimumab in the treatment of malignant melanoma. While anti-PD-1 antibodies were not available at the time this patient was treated, these drugs would certainly be considered in the treatment today, as monotherapy or in a combination regimen with ipilimumab based on benefit demonstrated in prospective clinical trials. Efficacy of anti-PD1 antibodies in patients with mucosal melanoma has been well demonstrated [26–29]. As D´Angelo *et al.* reported on 86 patients with mucosal melanoma treated in clinical studies including phase III trials with nivolumab monotherapy or in combination with ipilimumab objective response rates of 23.3% in patiens treated with nivolumab monotherapy and 37.1% in the combination arm. The median progression-free survival was 3.0 months in patients treated with nivolumab monotherapy and 5.9 months in patients treated with nivolumab combined with ipilimumab suggesting that nivolumab combined with ipilimumab have greater efficacy than either agent alone [27]. Effecacy of nivolumab in terms of irCR in patiens with mucosal melanoma has also been described [26] .

Vitiligo, affecting our patient, is a common adverse event of ipilimumab treatment reflecting T-cell responsiveness against melanocytic differentiation antigens in melanoma [30, 31].

In conclusion, the present case demonstrates that ipilimumab may be an effective treatment option in patients with metastatic mucosal melanoma, although, in general, mucosal melanomas compared to cutaneous melanomas were reported to have a less favorable outcome when treated with ipilimumab. Certain patients may derive a substantial benefit from ipilimumab therapy, but currently we have no reliable predictive biomarkers. Despite multiple effective treatment options for cutaneous melanoma, the data on treatment of melanomas of other primary sites are limited, and clinical decisions are often based on retrospective experience from anecdotal reports like the present case.
